# Data on quantification of signaling pathways activated by KIT and PDGFRA mutants

**DOI:** 10.1016/j.dib.2016.10.026

**Published:** 2016-11-02

**Authors:** Christelle Bahlawane, Martine Schmitz, Elisabeth Letellier, Karthik Arumugam, Nathalie Nicot, Petr V. Nazarov, Serge Haan

**Affiliations:** a Molecular Disease Mechanisms group, Life Sciences Research Unit, University of Luxembourg, Campus Belval, 6 Avenue du Swing, L-4367 Belvaux, Luxembourg; b Department of Infection and Immunity, LIH, 84 Val Fleuri, L-1526 Luxembourg; c Genomics Research Unit, LIH, 84 Val Fleuri, L-1526 Luxembourg

**Keywords:** c-KIT, PDGFRα, MAPK, PI3K, Gastro-intestinal stromal tumours, PD0325901, Stem cell factor

## Abstract

The present data are related to the article entitled “Insights into ligand stimulation effects on gastro-intestinal stromal tumors signaling” (C. Bahlawane, M. Schmitz, E. Letellier, K. Arumugam, N. Nicot, P.V. Nazarov, S. Haan, 2016) [1]. Constitutive and ligand-derived signaling pathways mediated by KIT and PDGFRA mutated proteins found in gastrointestinal stromal tumors (GIST) were compared. Expression of mutant proteins was induced by doxycycline in an isogenic background (Hek293 cells). Kit was identified by FACS at the cell surface and found to be quickly degraded or internalized upon SCF stimulation for both Kit Wild type and Kit mutant counterparts. Investigation of the main activated pathways in GIST unraveled a new feature specific for oncogenic KIT mutants, namely their ability to be further activated by Kit ligand, the stem cell factor (scf). We were also able to identify the MAPK pathway as the most prominent target for a common inhibition of PDGFRA and KIT oncogenic signaling. Western blotting and micro-array analysis were applied to analyze the capacities of the mutant to induce an effective STATs response. Among all Kit mutants, only Kit Ex11 deletion mutant was able to elicit an effective STATs response whereas all PDGFRA were able to do so.

**Specification Table**

**Table:** 

Subject area	Cancer Research
More specific subject area	Signal transduction AND Receptor Tyrosine Kinases
Type of data	Western blot; qPCR, FACS, micro-array, Computational modeling
How data was acquired	Fusion-FX7 chemiluminescence detection device (Vilber) for Western blotting,
	CLARIOstar microplate reader (BMG LABTECH) for fluorescence measurements of cell viability
	GeneChip Human Gene ST 2.0 arrays (Affymetrix) for micro-array
	FACS CantoII Instrument (Becton Dickinson, Heidelberg, Germany) for flow cytometry
	Model building and refinement with CHARMM
Experimental features	KIT and PDGFRA mutants were expressed in hek293 cell lines upon doxycycline addition
	Experiments were performed with/without ligand induction
Data source location	LIH, Luxembourg and university of Luxembourg, Luxembourg
Data accessibility	Data are available in the article, and at ArrayExpress E-MTAB-4548

**Value of the data**

• Expression of different mutant proteins (Kit and PDGFRA) in an isogenic background to allow a direct comparison of their signalling capacities, without the complex patient specific- background. The constructs and/or the Hek293 cell lines, could be used for further molecular characterization, protein-protein interaction experiments or protein localization studies. Identified mutations in GIST could easily be investigated by insertion of the newly discovered mutation in the WT constructs by site-directed mutagenesis. Plasmids are available upon request.
• Identification of a new feature specific to Kit mutants: their ability to be further stimulated by their natural ligand, in addition to their constitutive activation derived from the mutations observed in GIST.
• A new MEK inhibitor (PD0325901) was identified to be efficient in inhibiting GIST cell proliferation in the nanomolar range.

## 1 Data

The data presented here derived mainly from western blot analysis for the quantification of signaling pathways activated by KIT and PDGFRA mutants, from micro-array analysis for quantification of changes in gene expression levels induced by the different mutations and from flow cytometry analysis for the detection of KIT at the cell surface.

**Fig. 1:**
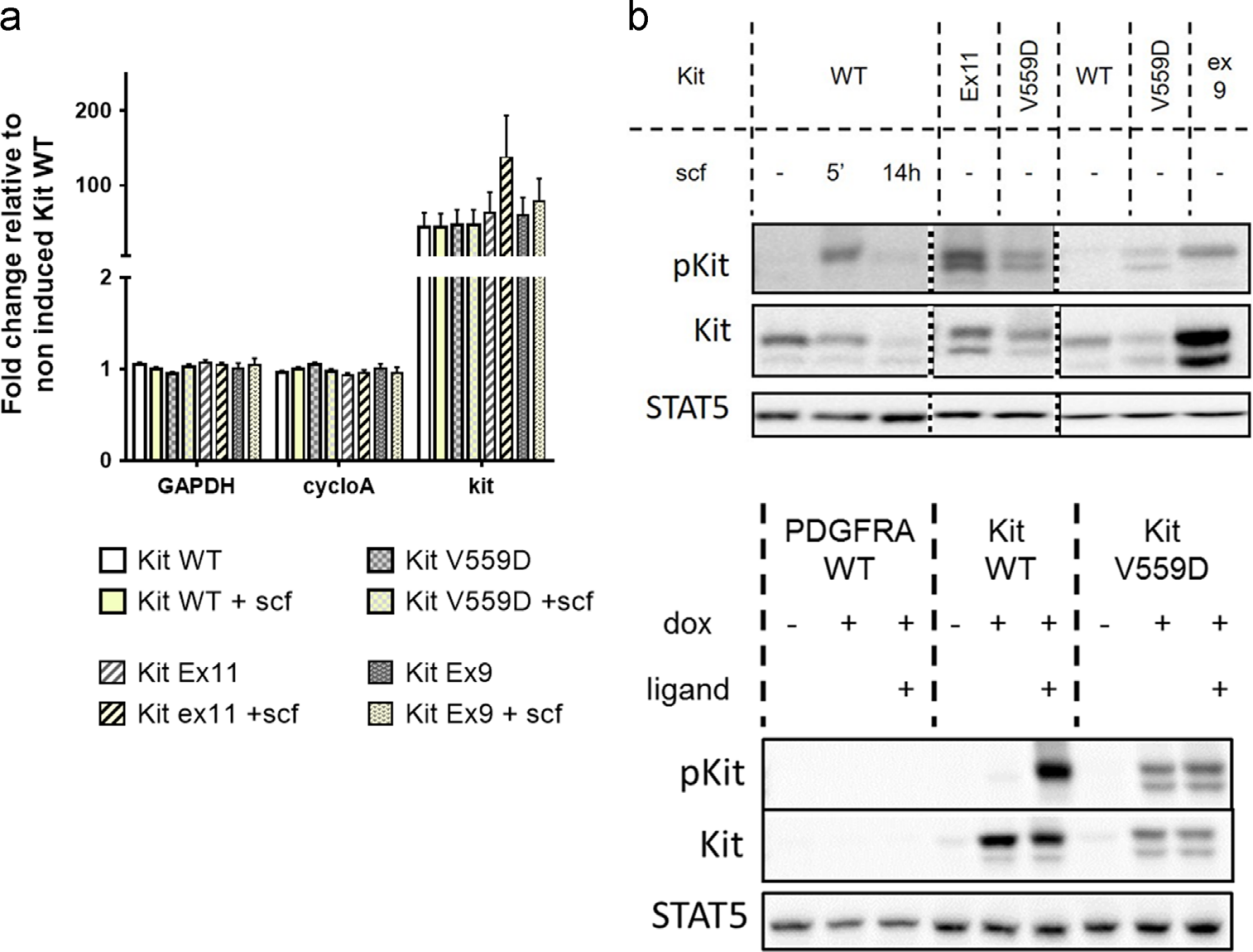
KIT RNA and protein expression levels in the stable transfected cells. (a) KIT mRNA expression level as assessed by qPCR in Hek293 cells expressing KIT WT, KIT Ex11, KIT Ex9 and KIT V559D 14 h after induction with doxycycline (5 ng/ml). SCF was added at 100 ng/ml for the time of induction. Data represent the means of 3–6 biological replicates and are normalized using Genorm following the MIQE guidelines [2]. (b) Western blot analysis indicating KIT expression level as well as phosphorylation status in Hek293 cells expressing KIT WT, KIT Ex11, KIT Ex9 and KIT V559D 14 h after induction with doxycycline (5 ng/ml). SCF was added at 100 ng/ml for the time of induction or 5 min before cell harvesting as indicated on the figure. Representative data of 3 biological replicates. STAT5 is used as loading control.

Doxycycline induces Kit expression by a factor 100 in all cell lines and comparable mRNA expression levels were observed for all mutants (Fig. 1a). However, some differences in the protein expression levels were observed between the different constructs (Fig. 1b).

**Fig. 2:**
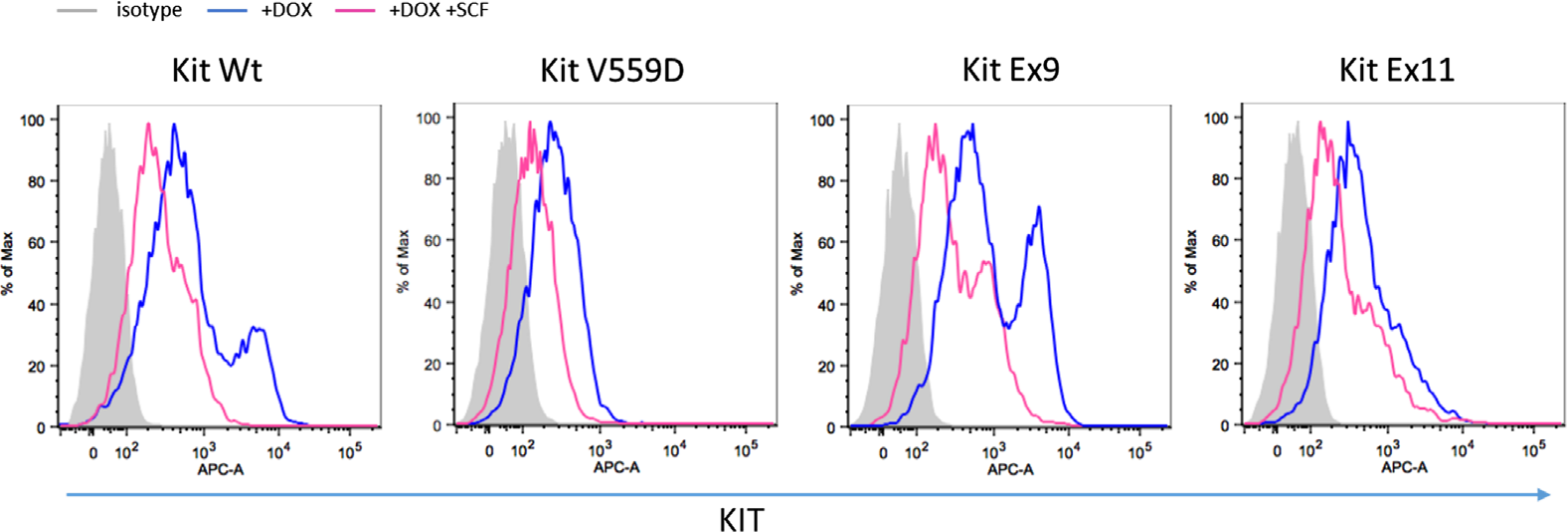
Effect of ligand stimulation on KIT expression at the cell surface.

The ratio of surface (Fig. 1a in [1]) to total KIT expression (Fig. 1b in [1]) indicates that KIT WT is expressed almost exclusively at the surface, while this is the case for 70% of KIT Ex9. This value drops to 50% for both Ex11 mutants. The results of the FACS analysis after SCF stimulation (Fig. 2) indicate the decrease of KIT expression at the cell surface for all KIT mutants and wild type following stimulation with KIT ligand, SCF.

The Mean Fluorescence Intensity (MFI) of Kit staining in the different Kit mutants are indicated in Table 1.

**Fig. 3:**
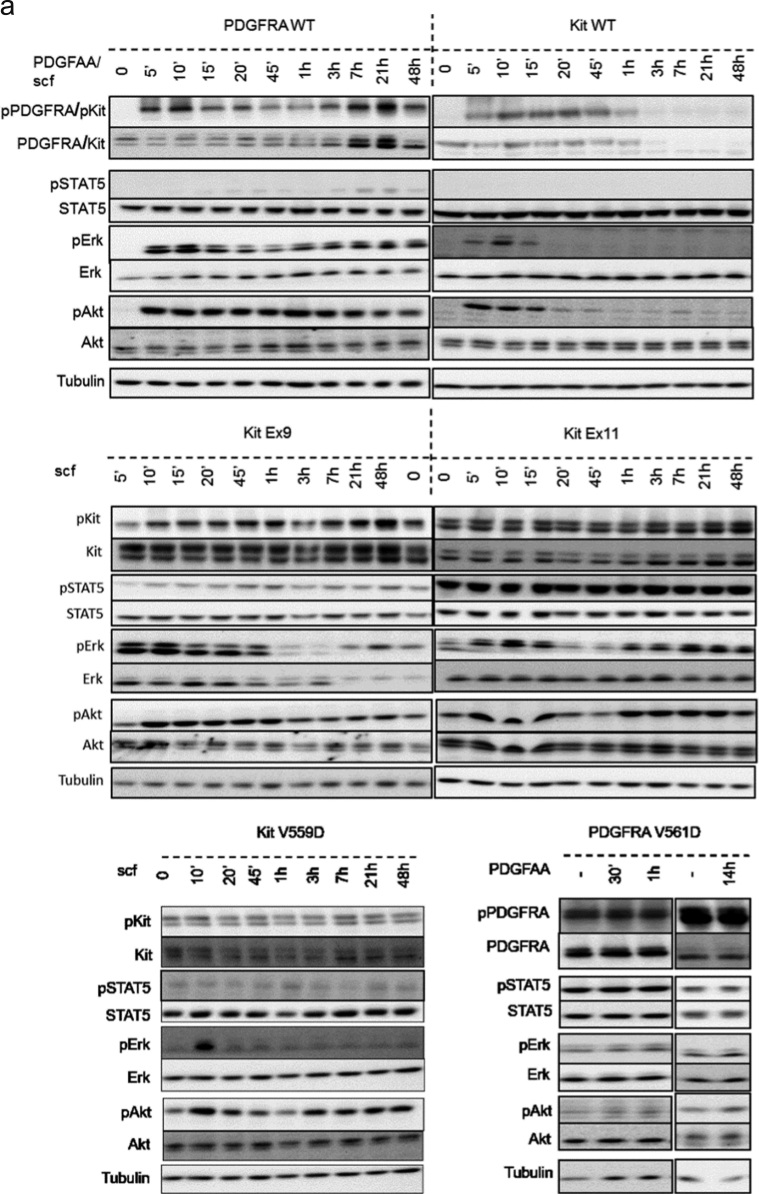

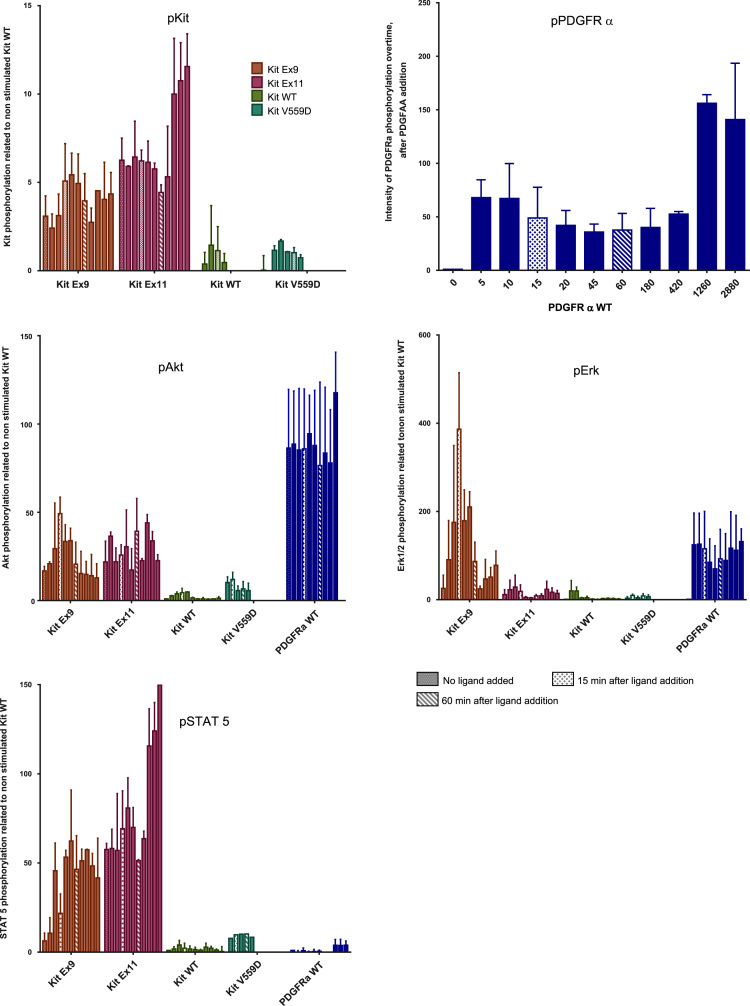
Effect of ligand stimulation on downstream signaling in GIST mutants. (a) Western blot analysis indicating the phosphorylation status of KIT/PDGFRα, STAT5, Akt and Erk in Hek293 cells expressing PDGFRα WT, PDGFRα V561D, KIT WT, KIT Ex11, KIT Ex9 and KIT V559D after PDGFAA or SCF addition. Representative blots of 3 biological replicates. Tubulin is used as loading control. (b) Quantification of the signal intensities from the western blots shown in **a**. Data were calibrated using the sample “KIT WT non-stimulated” (background level), except for PDGFRA phosphorylation where PDGFRa WT non-stimulated was used. Each dot represents the mean of biological triplicates and the error bars the standard error of the mean. From left to right, bars represent the signal intensities after ligand addition (1st bars correspond to no ligand, 15 and 60 min after ligand addition are marked for better visibility).

While PDGFRα, Akt, and Erk phosphorylation were induced by PDGFAA in PDGFRaWT, phosphorylation of PDGFRα, Akt, Erk and STAT5 remained identical to the non-stimulated control for the PDGFRA mutant V591D, as previously shown [3]. In contrast, Kit mutants exhibit all constitutive phosphorylation of kit at tyrosine 703 but the signal intensities for Erk, Akt and STAT5 phosphorylation was further increased upon SCF stimulation (Fig. 3a and Fig. 3b).

**Fig. 4:**
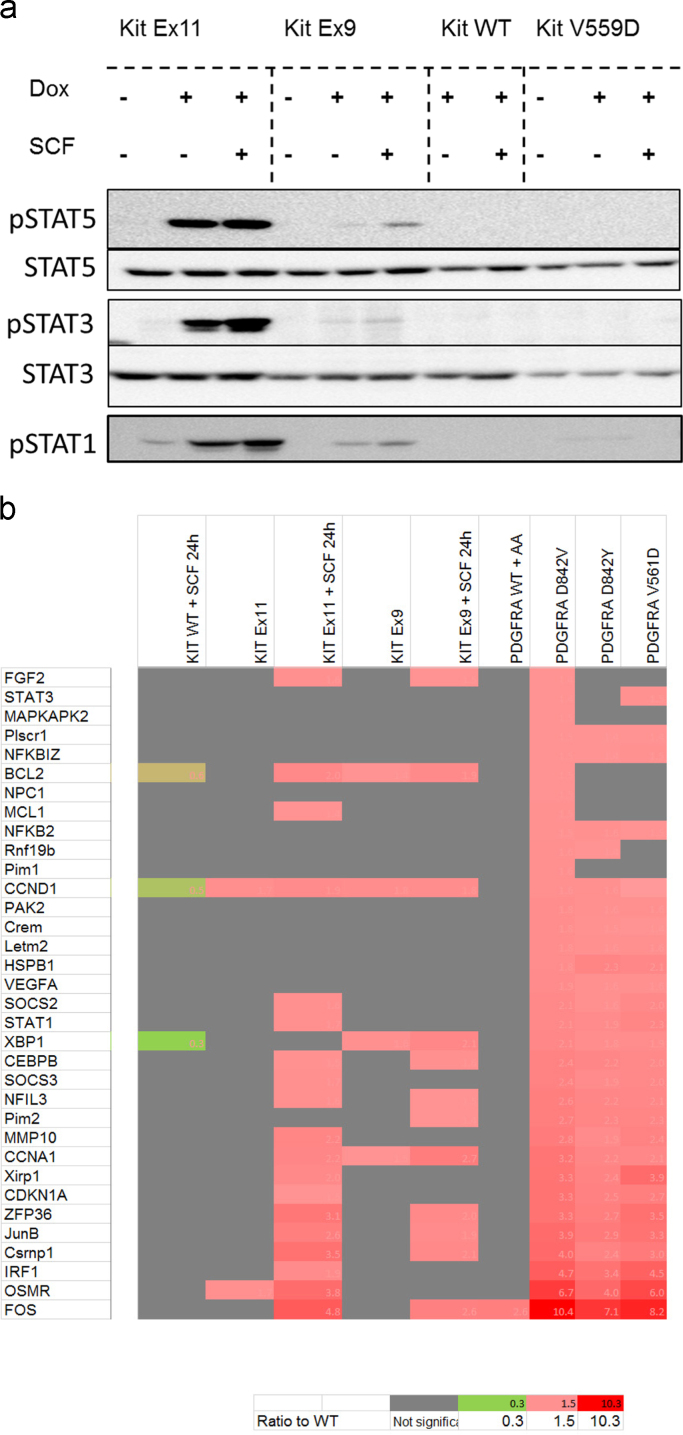
Activation of STAT species by GIST mutants. (a) Nuclear translocation of STAT species. Nuclear extracts were prepared as previously done [4], diluted in 4 times Laemmli buffer and subjected to Western blot analysis. Phosphorylation status of STAT5, STAT3 and STAT1 is shown for nuclear extract prepared from Hek293 cells expressing KIT WT, KIT Ex11, KIT Ex9 and KIT V559D. (b) Induction of known STAT target genes by GIST mutants. Gene expression level of known STAT target genes, previously identified to be induced in PDGFRA GIST mutants [3], was retrieved from micro-array data and presented as heat map. Grey boxes indicate that the genes are not part of the DEG list for the corresponding mutant (FDR>0.05 or AbsFC<0.5). The intensity of the red color corresponds to the value of the ratio to the background (Hek293 KIT WT non-stimulated for KIT WT and mutants and Hek293 PDGFRα non-stimulated for PDGFRα mutants). (For interpretation of the references to color in this figure legend, the reader is referred to the web version of this article) .

STATs translocation to the nucleus was identified for Kit Ex11 deletion mutant and for Kit Ex9 duplication mutant to a lower extend. Induction of gene expression known to be part of the STAT pathway was found for Kit Ex11 deletion mutant after SCF stimulation as well as for Kit Ex9 duplication mutant upon SCF stimulation to a lower extend (Fig. 4a and Fig. 4b).

We investigated KIT Ex11 specific gene signature, comparing KIT Exon 11 deletion mutant regulated genes to other GIST mutants (PDGFRA mutants and KIT Exon 9). 277 genes (Table 3) were differentially expressed in KIT Ex11 deletion mutant only. As noted in [1], these genes were associated with “cell cycle” and “insulin signalling pathway”.

**Fig. 5:**
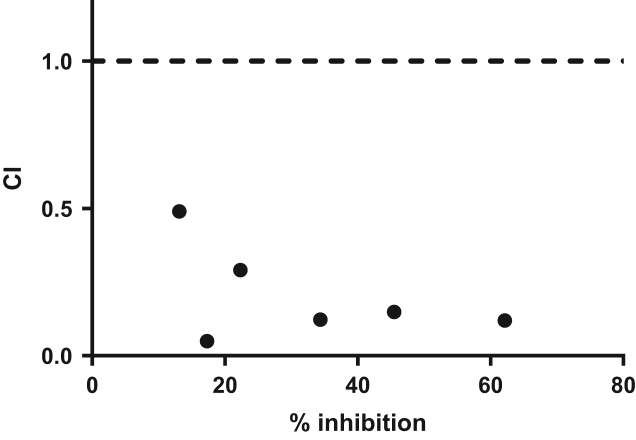
Synergistic assessment for PD0325901 and XL184 inhibitors in GIST882.

Both inhibitors were added to the medium 24 h after seeding for 30 h. Both drugs were added individually and in combination with a constant ratio of 1:1 (Fig. 5). Viability was assessed using PrestoBlue following the manufacturer׳s recommendation. Results were analysed using the Compusyn software [5] and the combination Index (CI) values are represented as a function of the percentage of inhibition. CI values below 1 indicates a synergy between the two compounds, while CI values above 1 indicates antagonism.

**Fig. 6:**
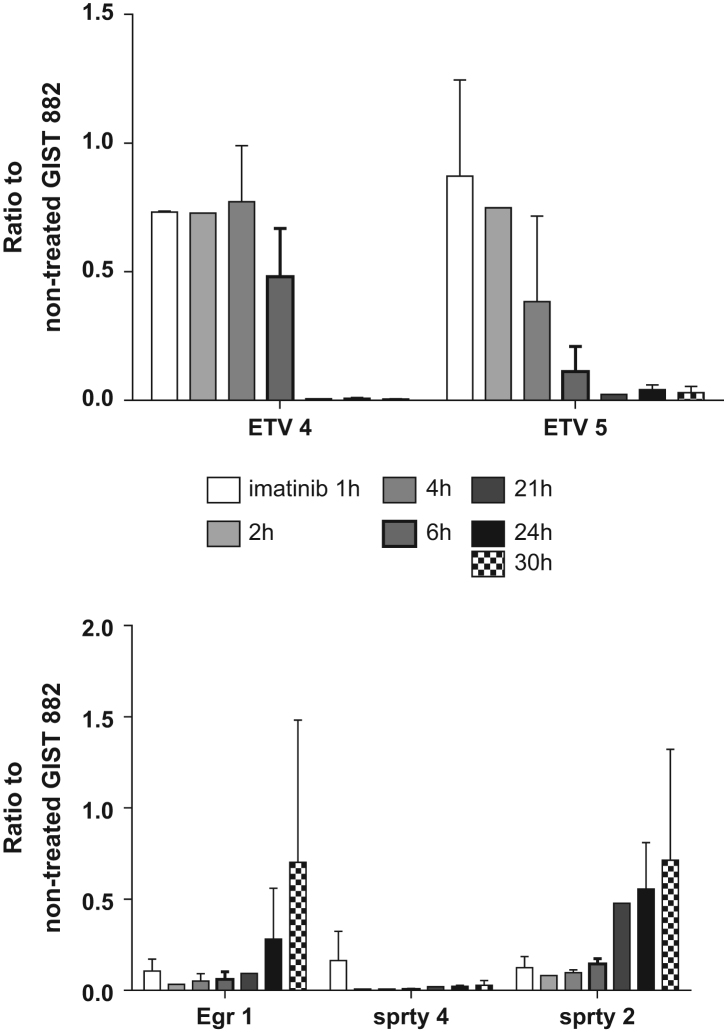
Imatinib effect on MAPK gene expression level over time.

GIST882 were treated with 100 nM imatinib for different times and mRNA expression level of ETV4, ETV5, egr1, FosL2 and FosB were assessed by qPCR. *n*= 3, Mean±SEM (Fig. 1, Fig. 2, Fig. 3, Fig. 4, Fig. 5, Fig. 6).

qPCR primers are listed in Table 2.

## 2 Experimental design, materials and methods

### 2.1 Flow cytometry analysis

Cell surface expression of KIT wild type and mutants was analyzed by flow cytometry using a FACS CantoII Instrument (Becton Dickinson, Heidelberg, Germany) either without ligand (blue lines) or 15 minutesmin after stimulation with 100ng/ml100 ng/ml SCF (pink lines). Cells were then either incubated with 10 μL KIT primary antibody (anti-CD117-APC conjugated; C7244; Dako, Belgium) for cell surface expression. Specificity was controlled using an isotype-matched/ APC conjugated antibody (grey).

### 2.2 Western blot analysis

Cell lysis was performed on ice, using 1x Laemmli buffer. Proteins were subjected to SDS-PAGE, transferred to polyvinylidene difluoride membranes (Roth) and probed with primary antibodies. Primary antibodies against PLCγ and phosphospecific antibodies against STAT1 (pTyr701), STAT3 (pTyr705), ERK1/2 (pThr202/pTyr204), PDGFRA (pTyr849)/β(pTyr857), AKT (pSer473) and Mek1/2 were purchased from Cell Signalling Technology. Anti-STAT1 and anti-STAT3 antibodies and phosphospecific antibodies for STAT5 (pTyr694) and PLCγ1 (pTyr783) were obtained from BD Biosciences. Antibodies against STAT5 (C-17), PDGFRα (C-20), ERK1 (C-16), ERK2 (c-14), AKT1/2 (N-19) and tubulin (DM1A) were bought from Santa Cruz. Anti-CD117 (KIT) antibody was obtained from Dako and horseradish peroxidase-conjugated secondary antibodies from Cell Signalling Technology. Signals were detected on a Fusion-FX7 chemiluminescence detection device (Vilber) using a home-made ECL (Enhanced ChemiLuminescence) solution containing 2.5 mM luminol, 2.6 mM hydrogenperoxide, 100 mM Tris/HCl pH 8.8 and 0.2 mM para-coumaric acid [6]. Signal intensities were quantified using the Bio1D analysis package (Vilber).

### 2.3 Microarray analysis

293FR cells expressing KIT-WT, KIT Ex11 deletion mutant and KIT Ex9 mutant were incubated with 5 ng/ml doxycycline and 100 ng/ml SCF for 21 h in DMEM with 1%FCS. Cells were then starved for 3 h (without FCS) and further stimulated with SCF. RNAs of three biological replicates were isolated using the miRNeasy Mini KIT (Qiagen) according to manufacturer׳s instructions with additional on-column DNase I digestion. RNA quality and purity were assessed using a Nanodrop Spectrophotometer (Thermo Scientific) and Agilent 2100 Bioanalyzer (Nano KIT). Gene Expression analysis was performed using GeneChip Human Gene ST 2.0 arrays (Affymetrix). Quality control and data normalization were performed as previously reported [3], [7]. We focused on differentially expressed genes (DEG) across the mutants comparing with non-stimulated KIT-WT. To exclude non-relevant lowly expressed transcript clusters, only those showing log2 expression above 4.5 were kept for further analysis. Transcript clusters were further summarized in order to obtain a single expression value for each gene in each experiment. The differentially expressed genes were statistically evaluated by two-factor linear model with empirical Bayes statistics approach using limma package of R/Bioconductor [8]. In order to correct for the false discovery rate (FDR), the Benjamini and Hochberg step-up method correction was applied. Probe-sets with FDR <0.05 and absolute fold change >0.5 were considered to be significantly differentially expressed (DEGs). Microarray data are available in the ArrayExpress database (www.ebi.ac.uk/arrayexpress) under accession number E-MTAB-4548. KIT Ex11 specific gene signature was determined by comparing KIT Exon 11 deletion mutant regulated genes to other GIST mutants (PDGFRA mutants and KIT Exon 9). 277 genes.

### 2.4 Nuclear extract preparation

Nuclear extracts were prepared as previously done [4]. In brief, cells were washed with ice-cold PBS, harvested gently with cell scraper and centrifuged at 4 °C for 5 min, 4000 rpm. The pellet was then resuspend in Buffer A (10 mM Hepes/KOH pH7.9, 1.5 mM MgCl2 and 10 mM KCl). Following 10 min incubation on ice, samples were centrifuged at maximum speed, 4 °C for 5 min. The operation was performed a second time and the pellets were finally resuspended in Buffer C for nuclear extraction (20 mM Hepes/KOH pH7.9, 420 mM NaCl, 1.5 mM MgCl2, 0.2 mM EDTA and 25% glycerol). Protein concentrations were determined using Bradford reagent before analysis by western blotting.

### 2.5 Synergy analysis

Synergy between Receptor Tyrosine kinase (RTK) and MEK inhibitors were performed at constant ratio, as recommended by Chou et al. [5], [9]. GIST primary cells were seeded in 96 well plates 24 h prior treatement. Inhibitors were added at concentration between 5 nM and 13 μM, either alone or in combination to a final volume of 90 μL. Endpoint viability was assessed using PrestoBlue (Thermofischer), by adding 10 μL of reagent to each well. Following 30 min incubation, fluorescence intensities were recorded on a CLARIOstar microplate reader (BMG LABTECH). Inhibition was then calculated as a ratio to the non-treated samples.

## Figures and Tables

**Table 1:** Mean Fluorescence Intensity (MFI) of Kit staining in the different Kit mutants.

MFI	Kit WT		Kit V559D		Kit Ex9		Kit ex11	
	−SCF	+SCF	−SCF	+SCF	−SCF	+SCF	−SCF	+SCF

Surface	2099	111	172	11	1435	284	562	222
Overall expression	2122	642	374	319	2106	1355	1008	852

**Table 2:** primers used for qPCR analysis.

Targeted gene	Primers sequences
GAPDH	gtccttccacgataccaaagt
	atgagaagtatgacaacagcct
	
HPRT	tggacaggactgaacgtctt
	gagcacacagagggctacaa
	
PPIA/cycloA	cagacaaggtcccaaagaca
	ccattatggcgtgtgaagtc
	
Tubulin	agatcggtgccaagttctg
	ccacctgtggcttcattgta
	
ETV4	gcccctcgactctgaagat
	tggaaatcaggaacaaactgc
	
ETV5	atccccgattatactttgacg
	agaagggtgaccaggaactg
	
Kit	acaaagagcaaatccatccc
	tgtaggtcagaatcatcacaataat
	
egr1	agtggtttggctggggtaa
	ctacgagcacctgaccgc
	
sprty2	ttgcacatcgcagaaagaag
	ggtcactccagcaggcttag
	
sprty4	gggagccactgagaacagag
	tggctcctaaatccatcctg

**Table 3:** KIT Ex11 deletion mutant specific DEG, with FDR<0.05 and absolute logFC>0.5.

ACER2	CCNYL1	EYA1	IRX4	LOC401321	NKX3-1	RN5S217	SNORA10	UTRN
ADAMTS20	CD109	FAHD1	ITGA2	LOC642838	NLRP1	RN5S335	SNORA16B	VRK3
ADCK3	CD3EAP	FAM46A	JAM2	LOC645166	NOA1	RND2	SNORA2A	WDR63
ADCY1	CD68	FAM47A	JMY	LPAL2	NR1D2	RNF167	SNORD111	WDR77
AKAP8	CDC25B	FAM59A	KCNA3	LPCAT2	NRN1	RNFT1	SNORD126	WWP2
ALDH1L2	CDT1	FAM64A	KDELR1	LPHN3	OLIG2	RNU2-7P	SNORD3C	ZC3H6
ALG12	CES3	FAM83B	KDELR3	LPPR4	PABPC1L	RNU7-6P	SNORD60	ZFP14
AMIGO2	CHEK2	FBXO32	KIAA0355	LRFN1	PAMR1	RPL13P5	SNORD71	ZNF140
ANKRD18DP	CITED1	FLJ44342	KIAA1430	LURAP1L	PAQR4	RYR2	SNORD91A	ZNF17
ANKRD20A12P	CKMT1A	FLJ45248	KIAA1609	LYSMD2	PARK2	SAMD15	SRCRB4D	ZNF239
ANKRD20A5P	CNN2	FRAT2	KLF11	MALAT1	PARM1	SCARA5	TAS2R31	ZNF280B
ANKRD27	CNTNAP3B	GALNT6	KLHL24	MCL1	PARP14	SCARNA21	TBX15	ZNF296
ARHGAP20	CPEB4	GGA2	KLHL36	MCM5	PARP4	SEMA6A	TCF25	ZNF347
ARHGAP35	CPPED1	GLTSCR2	KLRAP1	MDM2	PBX1	SERPINB1	TFAP4	ZNF485
ARHGEF1	CRKL	GRAMD4	LCMT1	MED12L	PCDH7	SGK1	THOC6	ZNF502
ARNT2	CRYBB2P1	GSPT2	LIG4	MGARP	PDP1	SGSM3	TIGD4	ZNF574
ASRGL1	CSDA	GYPC	LIN37	MGST1	PDPR	SHKBP1	TMEM143	ZNF581
BACE1-AS	CYP2S1	GYS1	LINC00282	MIR22HG	PHKB	SHMT2	TMEM159	ZNF70
BBIP1	CYP4×1	H2BFXP	LNP1	MIR3143	PKIA	SLC35B2	TMEM185B	ZNF738
BCL6	DDTL	HIST1H2BG	LOC100130776	MIR338	PLA2G7	SLC44A2	TMEM238	ZNF836
BEX5	DEDD2	HIST1H3A	LOC100132439	MIR3671	PLXNA2	SLC45A3	TNFRSF10D	ZSCAN12P1
BRD2-IT1	DLC1	HIST1H3H	LOC100133985	MIR4263	PMFBP1	SLC6A6	TNFRSF13C	
BZW1	DMRTA1	HLA-DRB5	LOC100272228	MIR4324	PPP1R13L	SLC7A6OS	TNRC6B	
C10orf10	DYNLL2	HSDL1	LOC100287628	MIR4530	PPP1R14C	SLC8A1	TOM1	
C10orf25	EBF3	IER5	LOC100288018	MIR4773-2	PROX1	SLC9A1	TP53I13	
C19orf54	ECH1	IFITM3	LOC100288520	MIR548A2	PRPH	SLC9A2	TRBV23OR9-2	
C22orf13	EID3	IGHD2-21	LOC100507299	MRI1	PRR12	SLC9A9	TRBV6-9	
C2orf77	EIF1	IL11	LOC147670	MTRF1L	PSEN2	SMOC1	TRPC1	
CABIN1	ELAC1	ILDR2	LOC147727	MTSS1	RAB39B	SMPD1	TRPS1	
CABLES1	EMR2	IMPA2	LOC284648	NFIX	RBL2	SNAR-D	TSC2	
CAMLG	EPHX4	INPP5D	LOC399815	NIPSNAP1	RHBDF1	SNAR-H	TSSK3	
CAP2	EPS15L1	IQCH	LOC400927	NIPSNAP3A	RN5S180	SNN	TXNDC17	
